# Diagnostic Performance of Breast Magnetic Resonance Imaging in Non-Calcified Equivocal Breast Findings: Results from a Systematic Review and Meta-Analysis

**DOI:** 10.1371/journal.pone.0160346

**Published:** 2016-08-02

**Authors:** Barbara Bennani-Baiti, Nabila Bennani-Baiti, Pascal A. Baltzer

**Affiliations:** 1 Department of Pharmaceutical Chemistry, University of Vienna, Vienna, Austria; 2 Department of Biomedical Imaging and Image-guided Therapy, Vienna General Hospital (AKH), Medical University of Vienna, Vienna, Austria; 3 Division of Hematology, Mayo Clinic, Rochester, Minnesota, United States of America; Universitair Medisch Centrum Utrecht, NETHERLANDS

## Abstract

**Objectives:**

To evaluate the performance of MRI for diagnosis of breast cancer in non-calcified equivocal breast findings.

**Materials and Methods:**

We performed a systematic review and meta-analysis of peer-reviewed studies in PubMed from 01/01/1986 until 06/15/2015. Eligible were studies applying dynamic contrast-enhanced breast MRI as an adjunct to conventional imaging (mammography, ultrasound) to clarify equivocal findings without microcalcifications. Reference standard for MRI findings had to be established by histopathological sampling or imaging follow-up of at least 12 months. Number of true or false positives and negatives and other characteristics were extracted, and possible bias was determined using the QUADAS-2 applet. Statistical analyses included data pooling and heterogeneity testing.

**Results:**

Fourteen out of 514 studies comprising 2,316 lesions met our inclusion criteria. Pooled diagnostic parameters were: sensitivity (99%, 95%-CI: 93–100%), specificity (89%, 95%-CI: 85–92%), PPV (56%, 95%-CI: 42–70%) and NPV (100%, 95%-CI: 99–100%). These estimates displayed significant heterogeneity (P<0.001).

**Conclusions:**

Breast MRI demonstrates an excellent diagnostic performance in case of non-calcified equivocal breast findings detected in conventional imaging. However, considering the substantial heterogeneity with regard to prevalence of malignancy, problem solving criteria need to be better defined.

## Introduction

Breast is the most frequently affected organ by cancer in women [1]. Imaging plays a major role in secondary and tertiary prevention of breast cancer. Depending on whether healthy women are screened for breast cancer or whether assessment of symptomatic patients or screening findings is performed, mammography, breast ultrasound and percutaneous image-guided biopsies play a major role in diagnosis and to rule-out cancer [2–4]. However, these methods individually or in combination can yield inconclusive results, whereby the presence or absence of breast cancer is not clearly ascertained. Not everyone agrees on what qualifies as an equivocal finding. In clinical practice, a variety of results are usually classified as such: asymmetry without associated microcalcifications, architectural distortions and other ambiguous abnormalities such as multiple lesions, discrepancy between clinical symptoms and imaging findings, benign biopsy results with insufficient radiological-pathological concordance, lesions that could not be sufficiently localized during biopsy attempts, as well as scars. In these instances, an additional imaging-based diagnostic test would be most welcome. Microcalcifications are considered less problematic, since these lesions can be visualized by mammography and the workup of these lesions either by biopsy or follow-up imaging does usually not require additional imaging modalities.

MRI is considered by most to be one of the most sensitive imaging modalities for the detection of breast cancer [5,6]. Thus, breast MRI has for instance been shown to be a good imaging modality to exclude advanced nodal disease and to be helpful in the differential diagnosis of architectural distortions [7,8]. However, the effectiveness of breast MRI as a problem-solving tool remains controversial. Regular concerns are that the high sensitivity of MRI may not be high enough to rule-out malignancy and that MRI may associate with a high number of false positive findings as reflected by a low positive predictive value. As a result, there currently is a lack of clear recommendations on the application of MRI to resolving breast-imaging equivocality.

Consequently, the purpose of this systematic review and meta-analysis was to evaluate the performance of breast MRI for diagnosis of breast cancer in non-calcified equivocal breast findings. Or, put otherwise: can breast MRI rule-in or rule-out malignancy in non-calcified equivocal breast findings?

## Materials and Methods

### Search strategy

Two authors (BBB, PAB), one of them with 13 years of clinical experience in breast MRI, independently performed a systematic query of all full-text articles in the openly accessible PubMed database from 01/01/1986 up to 06/15/2015 (www.ncbi.nlm.nih.gov/pubmed/). Search terms were predefined as “breast MRI BI-RADS 0”, “breast MRI BI-RADS 3”, “breast MRI problem-solving” and “breast MRI equivocal”. A separate search was performed for each search term combination as indicated by the quotation marks above. Resulting titles/abstracts were analyzed for eligibility and full texts were retrieved, accordingly. Since no specific MeSH terms for this systematic literature study were identified, additional results were obtained by backward snowballing [9]. Results at every step were compared and discrepancies solved in a consensus review. If no consensus was reached, a third reader (NBB) served as an arbitrator.

### Eligibility criteria for study selection

Eligible were peer-reviewed studies applying the index test, dynamic contrast-enhanced breast MRI, as an adjunct to conventional imaging (mammography, ultrasound) to clarify unequivocal findings without microcalcifications in at least 20 human subjects. The reference standard for index test findings was defined as presence of histopathological sampling or imaging follow-up of at least 12 months. A diagnosis of cancer by the reference standard was considered a positive finding, absence of cancer as a negative finding. No language restrictions were applied.

### Data extraction and quality assessment

Two authors (BBB, PAB) independently extracted the following data: publication year, study design (retrospective/prospective), patient number and demographics, whether patient recruitment was consecutive, and indications for MRI examinations. Moreover, technical MRI parameters (field strength, coil, contrast medium dosage, and whether fat saturation was applied) were also collected. Index test (breast MRI) and reference standard (histopathology, follow-up) data were retrieved to fit a cross-tabulation with true or false positives (TP, FP) and negatives (TN, FN). Imaging results were called positive in case of BI-RADS 4 or 5 and negative in case of BI-RADS 1, 2 or 3. In case only a subpopulation of a study fulfilled the eligibility criteria, these specific data were extracted. Quality of studies and likelihood of bias were independently (BBB, PAB) evaluated, using *Quality Assessment of Diagnostic Accuracy Assessment* (QUADAS-2), an applet that assesses risk and bias in patient spectrum, reference standard, disease progression, verification, clinical review, incorporation, test execution, study withdrawals, and indeterminate results [10]. Any disparities in the findings were resolved by consensus. If no consensus was reached, a third reader (NBB) served as an arbitrator.

### Study outcome

Diagnostic parameters of breast MRI were defined as the study outcome. These were: sensitivity (TP/(TP+FN)), specificity (TN/(TN+FP)), positive predictive value, PPV (TP/(TP+FP)) and negative predictive value, NPV (TN/(TN+FN)). The influence of a series of covariates was determined by subgroup analysis.

### Statistical analysis

Analyses were performed using STATA 13.0 (StataCorp, College Station, TX, USA) and OpenMetaAnalyst 12.11.14 (http://www.cebm.brown.edu/open_meta/download). The possible presence of publication bias was further assessed by use of Begg's funnel scatterplot and calculated by Egger's test. In this setup, the plot allowed us to probe both for bias and systematic heterogeneity as a function of study size, whereby a symmetrical inverted funnel shape denotes a largely unbiased dataset [11].

Once our concerns of data bias were addressed, we used data from individual cross-tabulations to construct forest plots for sensitivity, specificity, positive predictive value (PPV), and negative predictive value (NPV). Data heterogeneity was analyzed using Cochran´s Q and I-squared statistics. We calculated to that effect Q as the sum of squared differences between the effects of individual studies and those pooled across all studies. Since Q can be affected by sampling size, we also calculated I^2^, the latter being thought to represent the percent of variability that it is relatively independent from sampling errors, and defined as $I^{2}=\left(\frac{Q-df}{Q}\right)\text{x }100\text{\%}$, wherein *df* represents Q's degrees of freedom [12]. Pooled estimates for breast cancer prevalence, sensitivity, specificity, PPV, NPV, and likelihood ratios were calculated by applying random effects models or bivariate analyses using maximum likelihood estimates, as appropriate. In this study setting, the likelihood ratio can be used to calculate the post-test odds from the pre-test odds of breast cancer. In addition, a bivariate summary Receiver Operating Characteristics (sROC) curve was calculated and meta-regression using random effects models was used to investigate sources of heterogeneity. To put the results into context with Bayes´ theorem, a Fagan´s nomogram and probability-modifying plots for positive and negative breast MRI results were constructed.

## Results

### Study design and reporting

**Fig 1:**
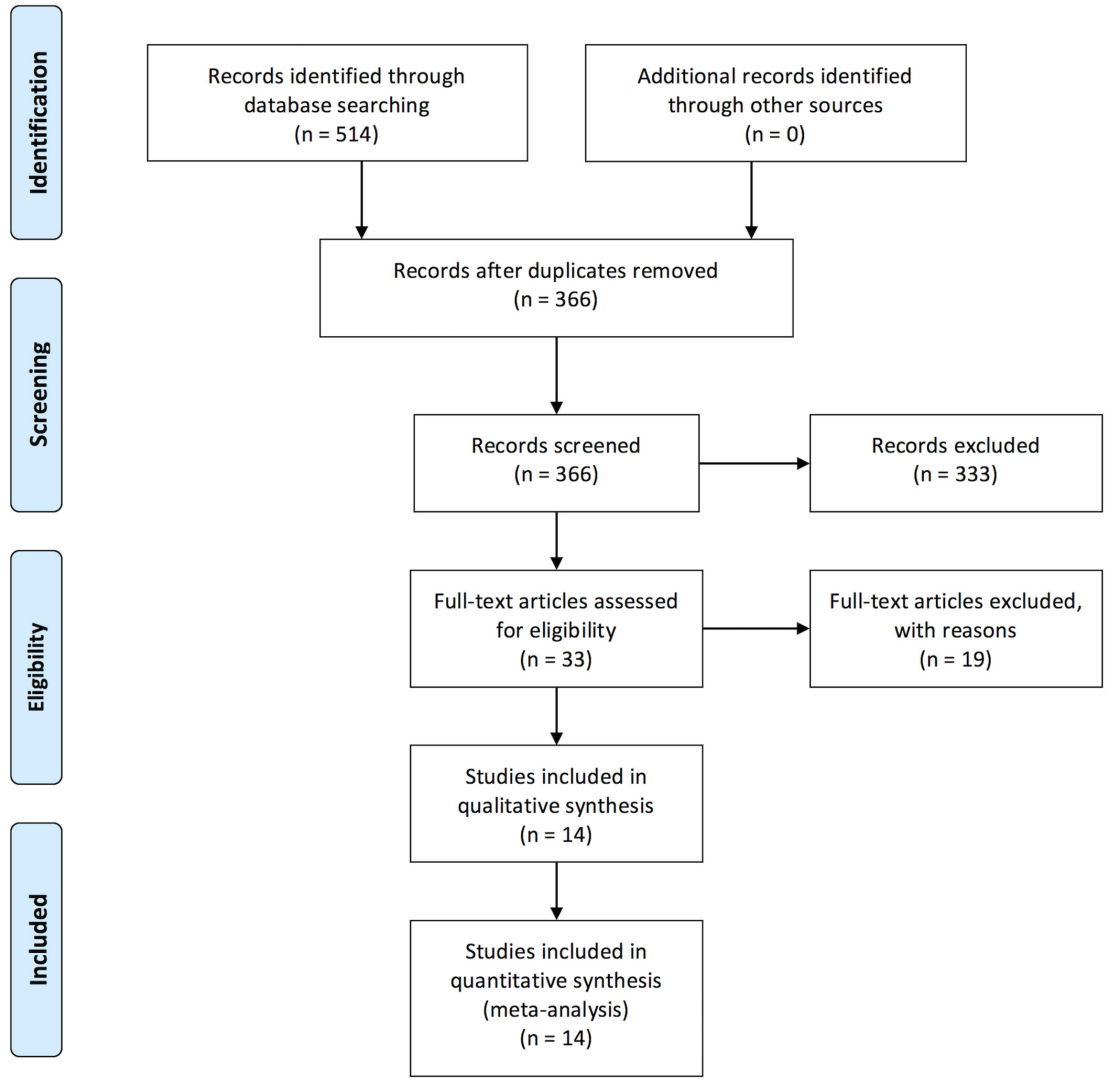
Flowchart depicting the selection process during systematic literature review.

Out of 514 peer-reviewed studies, we identified fourteen eligible reports comprising 2,316 cases [13–26]. In five of these fourteen studies, we extracted only data of the eligible subgroups [17,18,21,22,26]. This meta-analysis adheres to the *Preferred Reporting Items for Systematic Reviews and Meta-Analyses*, and Fig 1 depicts a PRISMA flow chart summarizing the selection process. The completed PRISMA checklist is given in S1 Table. Three of the included fourteen studies were prospective [14,20,26]. The remaining studies focused on retrospectively reviewing the diagnostic accuracy of breast MRI in a problem-solving population. Indications for MRI were described as required for further evaluation of unclear or suspicious findings, with or without questionable correlates in conventional imaging. Studies included primarily unclear BI-RADS 0 or 3 finding analogs, whereas some studies also included lesions labeled as BI-RADS 4. In the latter, however, the lesions could not be localized for biopsy and as such qualify as equivocal lesions [13,18,23]. Four studies indicated detailed reasons for referral to breast MRI (e.g. the exact type of clinical or imaging findings) [15,23,25,26], two of which stratified breast MRI results by indication [25,26]. The remaining ten studies did not specify detailed indications for breast MRI referral [13,16–22,24].

Nineteen studies were deemed not eligible due to various reasons, including lack of peer review, lack of extractable raw data, studies with too small of a sample-size (n<20), those focusing on the exclusion of malignancy in biopsy-proven lesions with uncertain malignant potential (B3) and recommended open surgery, those only aiming at diagnosing suspicious mammographic microcalcifications, or those limited to certain risk groups or specific clinical questions [27–45] (Fig 1).

All MRI technical data are summarized in S2 Table. QUADAS-2 analysis of risk of bias and applicability assessment identified a likely patient selection related bias in four studies: one recruited patients conditional on palpable findings [22], one on lesion size [20], and two considered solitary conventional BI-RADS categories only [14,26]. An unclear patient selection bias was assigned in another study due to restriction to two BI-RADS categories [17], and an unclear bias was also assigned in two studies since a proportion of the patients were lost to imaging follow-up [19,24]. Regarding applicability of the analyzed studies to the research question, bias was deemed low in the majority of studies. An assignment of unclear applicability bias was assigned to the two studies with patients lost to follow-up [19,23], and to one study investigating only BI-RADS 4 findings [26]. Detailed QUADAS-2 assessment results are given in S1 Fig.

**Fig 2:**
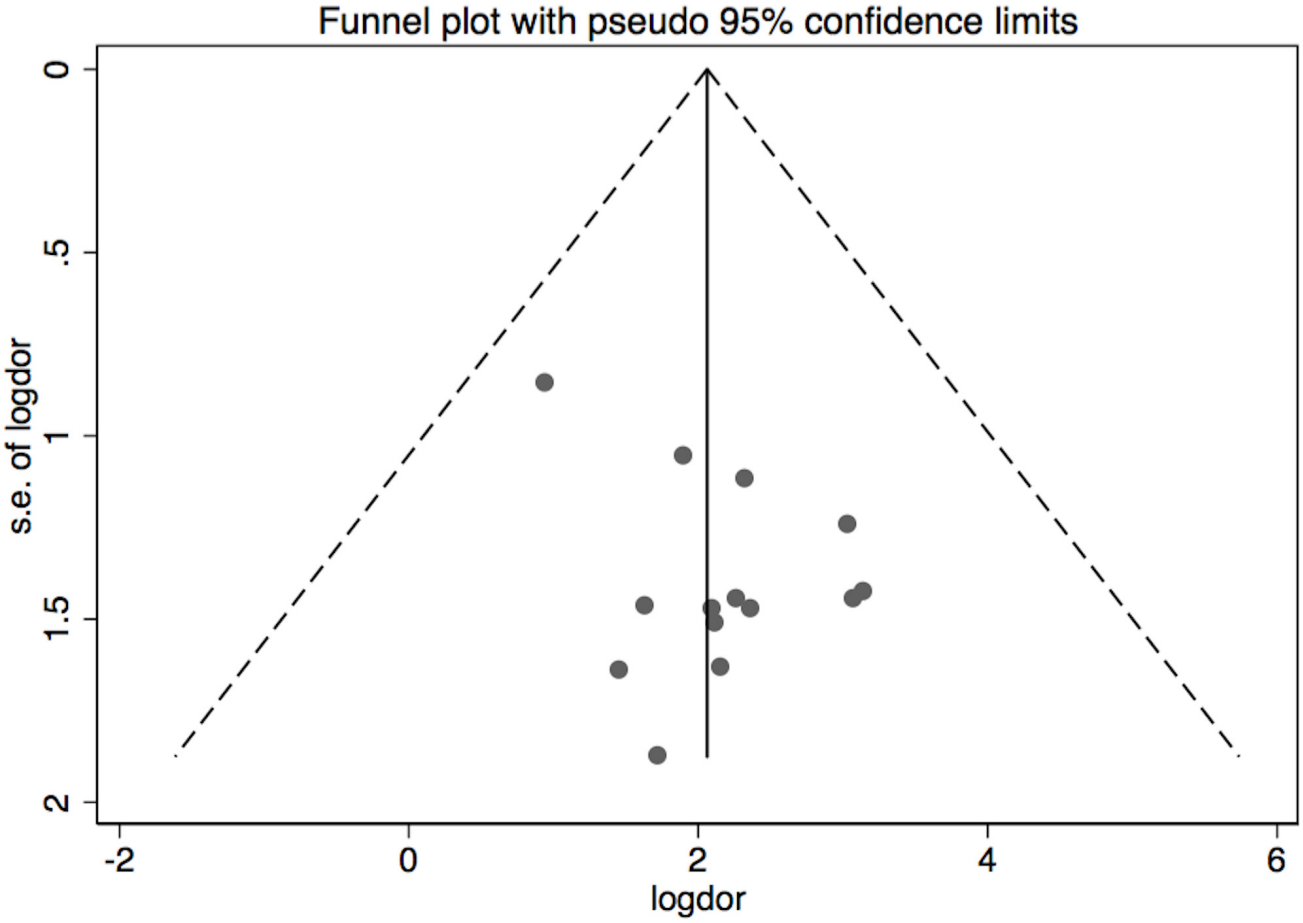
Begg's funnel scatterplot analysis of selected studies. The graph plots the logarithmic values of diagnostic odds ratios (l*ogDOR*) of considered studies in the abscissae axis against the standard error of *logDOR* in the ordinate axis. The two lines delimiting the inversed funnel denote pseudo 95% confidence intervals. Note the absence of any funnel plot asymmetry (confirmed by Egger´s testing).

Finally, a Begg's funnel plot analysis revealed a symmetrical distribution, indicating a lack of publication bias (Fig 2). The complimentary Egger's test also did not show a significant risk of publication bias (p = 0.12).

### Prevalence of malignancy and diagnostic performance of breast MRI

**Fig 3:**
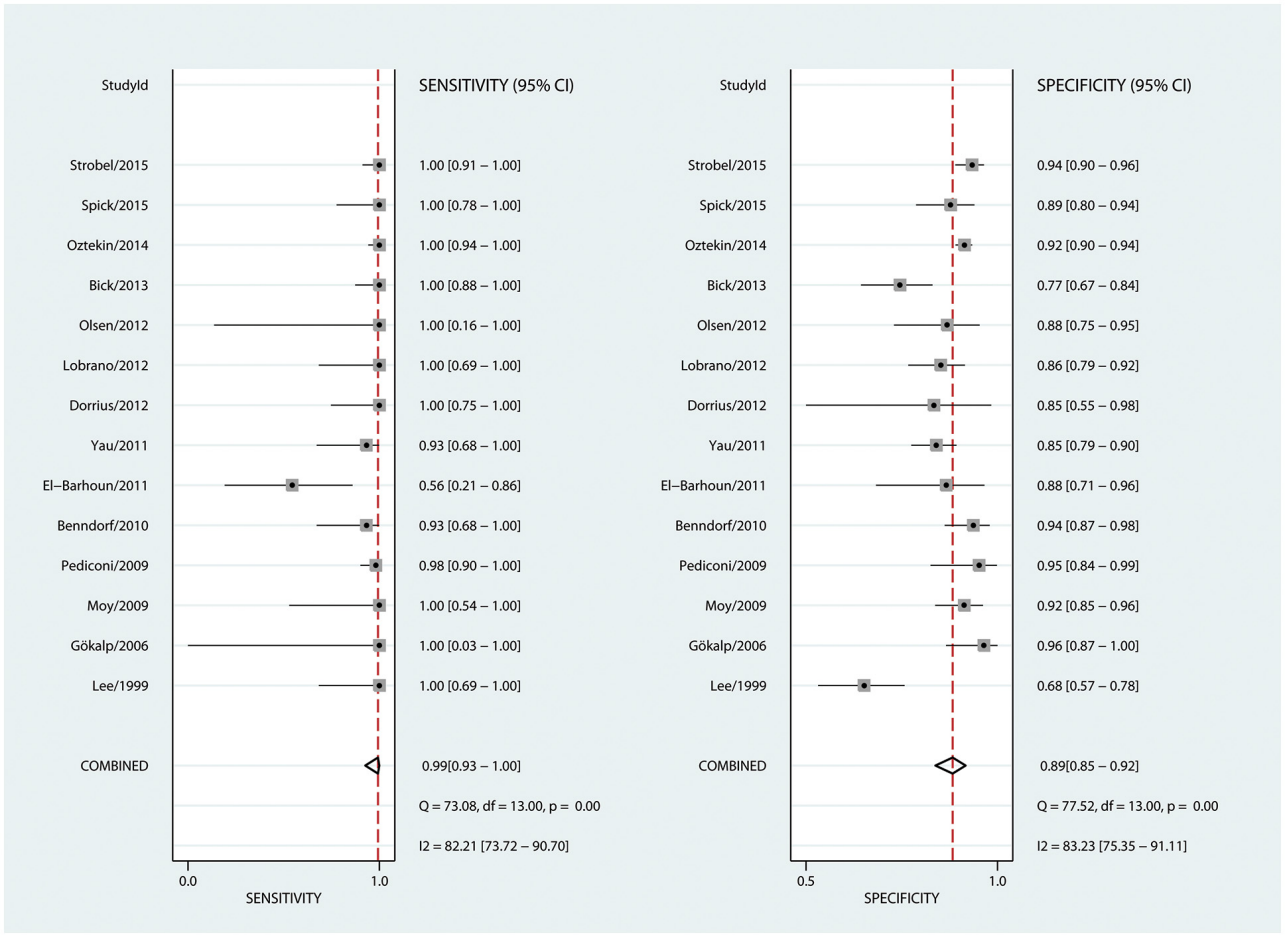
Forest plots of sensitivity and specificity. Sensitivity was defined as $\left(\frac{\text{tp}}{\text{tp}+\text{fn}}\right)$ and specificity as $\left(\frac{\text{tn}}{\text{tn}+\text{fp}}\right)$. tn: true negative; tp: true positive; fn: false negative; fp: false positive. All numbers have been rounded up or down to the closest second decimal.

**Fig 4:**
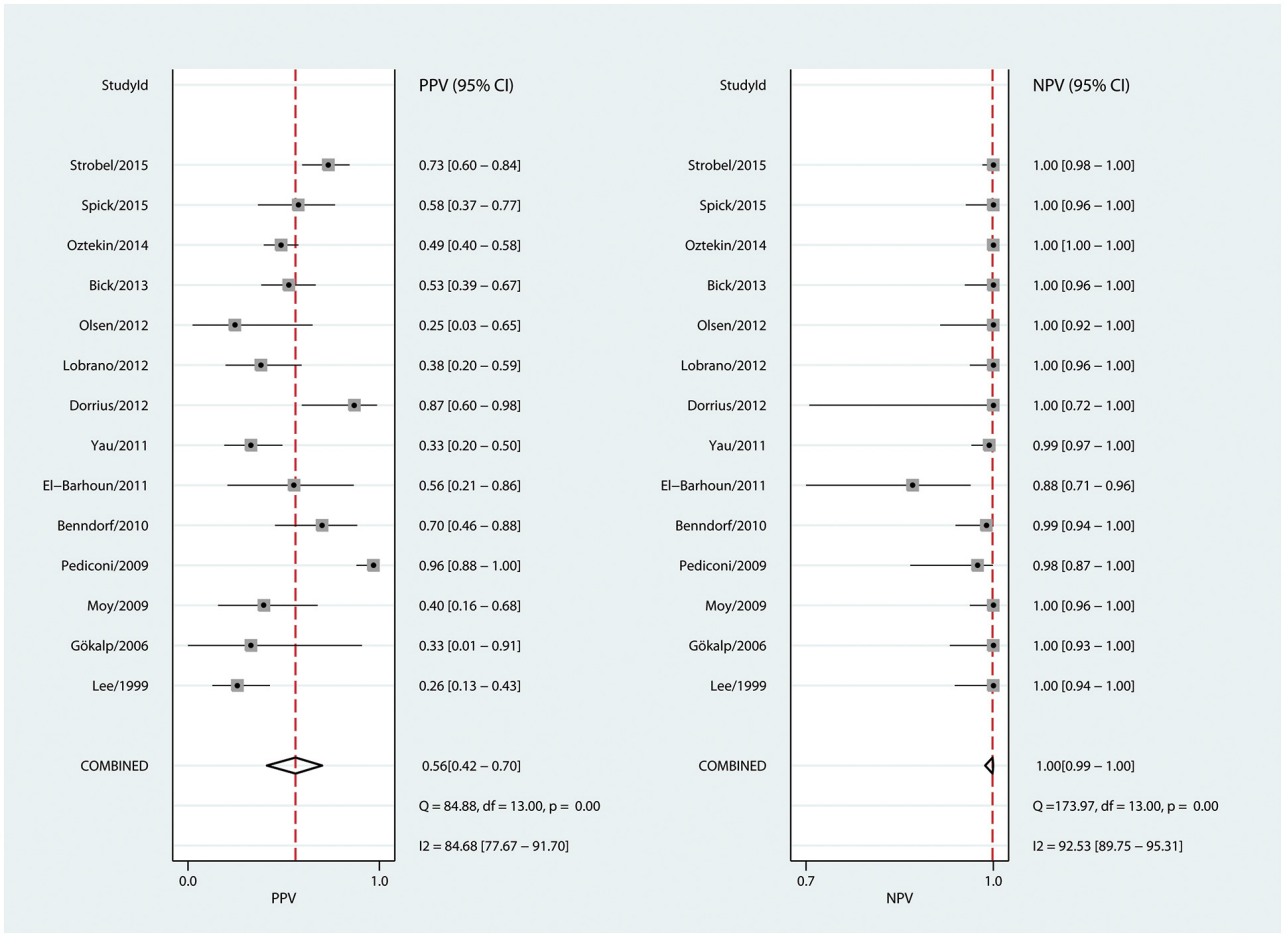
Forest plots of the positive and negative predictive values. The positive predictive value PPV was defined as $\left(\frac{\text{tp}}{\text{tp}+\text{fp}}\right)$ and the negative predictive value NPV as $\left(\frac{\text{tn}}{\text{tn}+\text{fn}}\right)$. tn: true negative; tp: true positive; fn: false negative; fp: false positive. All numbers have been rounded up or down to the closest second decimal.

**Fig 5:**
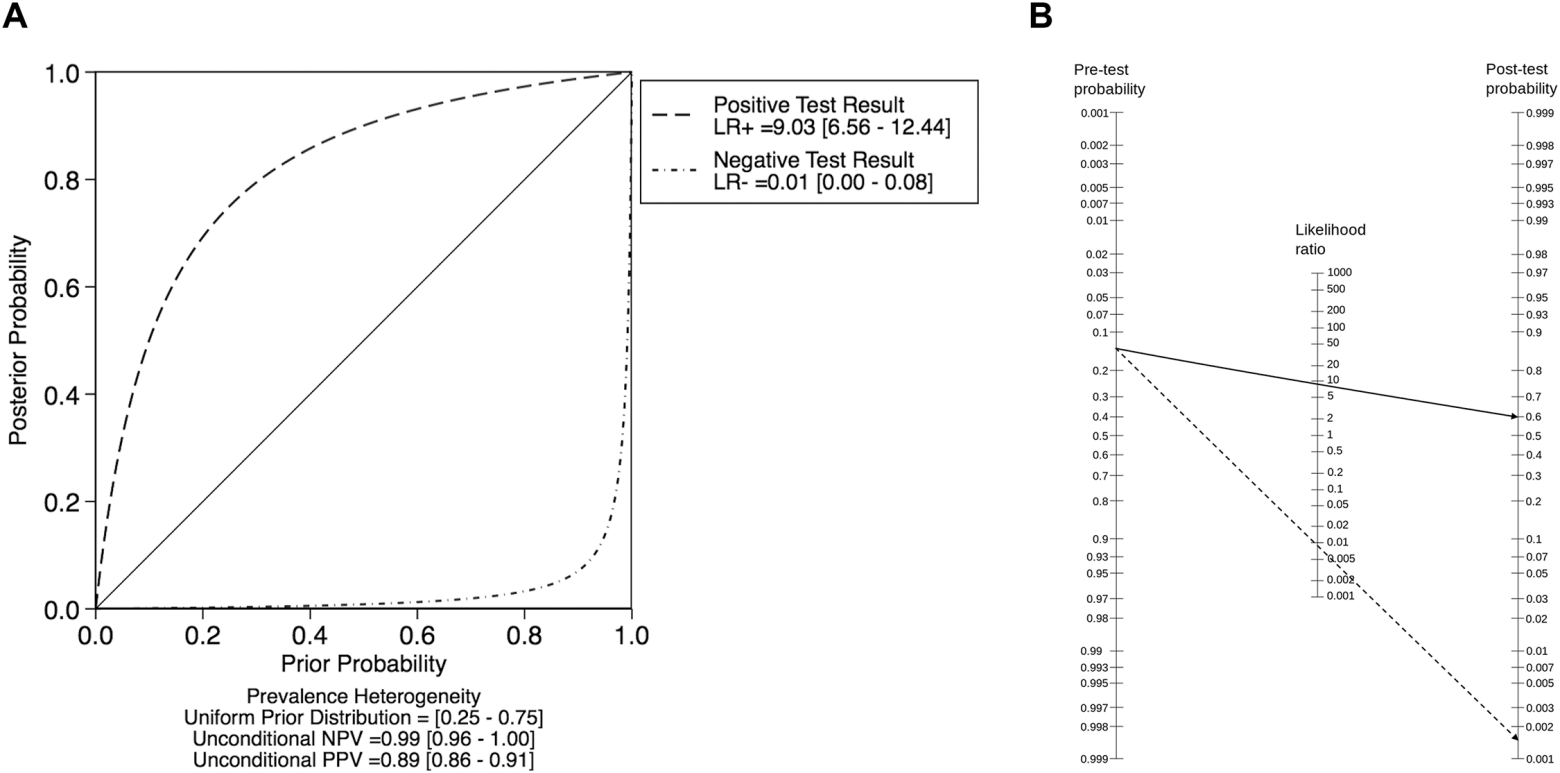
Pre- and post-test probabilities. **A.** Probability modifying plot. Note that post-test probabilities below 2% are achieved up to pre-test probabilities of 60%. **B.** Fagan´s Nomogram applying pooled positive (plain line) and negative (dashed line) likelihood ratios to a pretest probability of 25% (the 95% CI upper bond of pooled prevalence of malignancy in all selected studies). Resulting posttest probabilities were 60% and 0.15% for a positive or a negative MRI result, respectively.

Analysis of prevalence showed substantial heterogeneity ranging from 1.8 to 56.7% (Q 162.2, I^2^ 92%; p<0.0001, Table 1). Using a random effects model, pooled prevalence was 14.3% (95% CI: 9.8–18.8%). The number of true positives, false positives, true negatives, and false negatives in each study are listed in Table 2. A bivariate analysis sROC curve revealed an area under the curve (AUC) of 96% (95%-CI 94–98%, S2 Fig). We then run bivariate analyses of breast MRI-associated sensitivity, specificity, positive predictive value (PPV), and negative predictive value (NPV). The tested parameters also showed high heterogeneity as reflected by the corresponding I² and Q values. These were 82.2% and 73.1 (p < 0.001) for sensitivity, 83.2% and 73.5 (p < 0.001) for specificity, 84.7% and 84.9 (p < 0.001) for the PPV, and 92.5% and 174 (p = 0.001) for the NPV, respectively. Pooled sensitivity and specificity were high, reaching 99% (95% CI: 0.93–1) for sensitivity, and 89% (95% CI: 0.85–0.92) for specificity (Fig 3). PPV varied the most, ranging from 25% to 96.4% and yielding a pooled PPV of 56% (95% CI: 0.42–0.7; bivariate analysis; Fig 4). Based on pooled data, the likelihood ratio of a positive MRI scan was 9 (95% CI: 6.6–12.4, bivariate analysis; Fig 5), meaning that a positive MRI result increases the post-test odds of breast cancer by a factor of 9. Finally, with the exception of one outlier [18], the NPV ranged from 97.6 to 100%. Including this outlier, the pooled NPV was 99.9% (95% CI: 0.99–1.0, bivariate analysis; Fig 4). Altogether, seven false negative findings were described, four of which in the outlier study [18]. Three other studies reported a single false negative each [16,17,19]. There was only one false negative case, that of a Paget's disease of the breast, which showed no abnormality in mammography, ultrasound, and MRI [17]. Five of the remaining six false negatives were reader misinterpretations of abnormal MRI enhancements [16,18]. No specific MRI information was provided on the last false negative [19]. The pooled negative likelihood ratio was 0.01 (95% CI: 0.0–0.08; Fig 5), meaning that a negative MRI result decreases the post-test odds for breast cancer 100-fold. Applying Bayes´ theorem, malignancy can thus be ruled out up to pretest probabilities of 60%. Under this premise, the resulting likelihood of malignancy of a negative MRI is less than 2% (Fig 5). The malignancy likelihood of a negative breast MRI falls to below 1% if pretest probabilities are set to 40% (Fig 5). In our study population with a pooled prevalence of malignancy of 14.3%, the probability of malignancy in case of a negative MRI scan falls beneath 0.2%.

**Table 1:** Characteristics of patients and lesions considered in meta-analysis.

Author, year	QUADAS2 bias risk	Applicability concerns	MRI Indications**	Patients	Age	Ref. standard***	Lesions with FU only	FU months	Lesion size (mm)	Total cases	Malignancy prevalence
**Lee, 1999**	P	No	3	86	53 (30–80)	1	54	19 (5–66)	12.4	98	0.10
**Gökalp, 2006**	P	No	2	43	49.7 (37–68)	1	46	>24	9.2 (3–21)	56	0.02
**Moy, 2009**	P	No	3	115	54 (32–72)	1	100	34 (26–72)	16 (10–29)	115	0.05
**Pediconi, 2009**	P	No	3	97	47.5 (16–77)	1	NA	>18	NA	97	0.56
**Benndorf, 2010**	P	No	1, 2	113*	54.4 ±11,8	1	NA	>12	NA	113	0.12
**El-Barhoun, 2011**	P	No	3	41*	NA	1	8	>6	NA	41	0.12
**Yau, 2011**	P, R	No	3	204	NA	1	61°	12	NA	204	0.07
**Dorrius, 2012**	P	No	3	25	48.7 (32–69)	2	no FU	no FU	10-80^m^ 10-21^b^	26	0.50
**Lobrano, 2012**	P	No	3	66*	55 (20–89)	1	36°°	NA	NA	126	0.08
**Olsen, 2012**	P	No	3	51*	52 (31–84)	1	29	12	NA	51	0.04
**Bick, 2013**	P, R	No	3	135	57.3 (50–70)	1	52°°°	24	12 ≤10 16 >10	135	0.21
**Oztekin, 2014**	P	No	3	868	47 (16–82)	1	690	12	NA	868	0.07
**Spick, 2015**	P	No	1	111	51 (20–83)	1	NA	> 12	13.4 (6–44)^ml^ 23.8 (6–75)^nml^	111	0.14
**Strobel, 2015**	P	No	3	340*	53 (23–81)	1	218	> 18	NA	275	0.15
**Total**										**2,316**	**0.14**

P: patient selection; R: reference standard; H: histology; b: benign; m: malignant; FU: follow-up; NA: not available;

(*) patient subset fulfilling the selection criteria;

(**) 1 = BI-RADS 0, 2 = BI-RADS 3, 3 = other;

(***) 1 = histology and/or follow-up, 2 = histology only; m: malignant, b: benign, ml; mass lesions, nml: non-mass lesions

**Table 2:** Detailed indications for MRI, and extracted cross-tabulation data of MRI results against the reference standard.

Author, year	Indication for MRI	Total cases	True positives	False positives	False negatives	True negatives
Lee, 1999	Indeterminate significance of mammographic abnormality; ambiguous abnormality; lesion could not be located for biopsy; scar vs tumor at lumpectomy site or at benign biopsy site	98	10	28	0	60
Gökalp, 2006	BI-RADS 3	56	1	2	0	53
Moy, 2009	Further evaluation of inconclusive mammographic findings (asymmetry, architectural distortion, scar after benign biopsy)	115	6	9	0	100
Pediconi, 2009	Dense breast, problem solving, suspicious lesions	97	54	2	1	40
Benndorf, 2010	BI-RADS 0 and 3	113	14	6	1	92
El Barhoun, 2011	Suspicious imaging but no pre-MR diagnosis (30) or symptoms but mammogram/ultrasound unhelpful (11)	41	5	4	4	28
Yau, 2011	Problem solving (clinical, imaging, nipple discharge)	204	14	28	1	161
Dorrius, 2012	BI-RADS 3+4, lesion size ≥ 1cm	26	13	2	0	11
Lobrano, 2012	Problem solving (abnormal mammogram or ultrasound)	126	10	16	0	100
Olsen, 2012	Palpable masses with negative mammogram/ultrasound	51	2	6	0	43
Bick, 2013	Equivocal or minimal sign (single/multiple findings), lesion couldn’t be sufficiently localized during biopsy attempt, benign biopsy with insufficient radiological-pathological concordance	135	28	25	0	82
Oztekin, 2014	Problem-solving: BI-RADS 0, BI-RADS 1&2 on MG/US but clinical findings, BI-RADS 3,4	868	63	66	0	739
Spick, 2015	Problem solving	111	15	11	0	85
Strobel, 2015	Problem solving	275	41	15	0	219
Total		2,316	276	220	7	1,813

### Meta-regression analysis

Using random-effects models, multivariate meta-regression analysis was performed. A minor but significant influence on sensitivity was identified for the covariates case number and cancer prevalence (P<0.05, respectively). This tendency towards higher sensitivity in larger studies and studies with a higher prevalence of malignancy was no longer statistically significant after the outlier study by El-Barhoun and Pitman [18] was removed from the meta-regression (P>0.05, see S1 File). Meta-regression on factors influencing specificity showed a minor but significantly higher specificity in studies investigating lesions either classified as BI-RADS 0 and/or 3 as opposed to other or less defined indications (P = 0.024, S1 File).

## Discussion

According to our results, breast MRI demonstrates an excellent diagnostic performance in case of non-calcified equivocal breast findings detected at conventional imaging. In particular, and despite substantial heterogeneity, sensitivity and NPV were nearly 100%, and, consequently, the negative likelihood ratio was found to be very low. These findings indicate, that breast MRI can reliably exclude malignancy in most cases.

A regularly brought up argument against breast MRI is that of a low specificity and, subsequently low PPV. In other words, it is assumed that breast MRI causes unnecessary biopsies and costly diagnostic follow-up procedures. This argument is not *per se* wrong: while pooled specificity was found to be as high as 88% in our study, we were not able to extract data on short-term follow-up examinations initiated by breast MRI in the investigated setting. Therefore, the number of short-term follow-up examinations necessary to achieve the high observed specificity remains elusive. On the other hand, as shown in this meta-analysis, PPV values were within the acceptable range of PPV for conventional imaging [46]. In addition, it has been shown that the majority of MRI-detected findings can be further identified by targeted ultrasound, providing a cost-effective and broadly available means of biopsy and follow-up in MRI-positive cases [47].

Our high diagnostic performance indices warrant some comparison with prior reports. The most recent systematic review that queried the effectiveness of breast MRI in resolving equivocal findings dates to 2010. The study comprised five studies and a total of 376 lesions including both calcified and non-calcified breast lesions [48]. The studies including calcified lesions showed inferior results as compared to those investigating non-calcified lesions. As stated above, workup of microcalcifications either by biopsy or follow-up does usually not require additional imaging modalities. The NPV for non-calcified lesions was reported as 100% in that systematic review which is in line with our findings. Among 283 malignant lesions in our study, only seven were missed by MRI. Five out of these seven false negative findings were actually visible as abnormal enhancements but were misinterpreted by the reporting radiologists [16,18]. One was a Morbus Paget of the mammilla and one was not visible by MRI [17,19]. A prior meta-analysis on breast MRI did report somewhat lower pooled diagnostic estimates: sensitivity of 90% and specificity of 72% [49]. This prior work is actually quoted as a reason by the EUSOMA recommendations why a negative MRI does not exclude breast cancer [50]. The lower diagnostic performance reported by the referenced work is likely due to data pooling of diagnostic accuracy studies on breast MRI without a focus on specific indications nor study designs. In particular, the prior meta-analysis included exploratory studies, such as those on artificial neural networks, and data on single diagnostic criteria such as the “peripheral washout” or “blooming sign” that refer to cancer specific diagnostic criteria [49]. We think that most would agree that such single diagnostic criteria (e.g. lesion margins) are not representative of the diagnostic potential of an imaging method as radiological diagnosis relies on the empirical or algorithmic combination of multiple imaging findings. On the other hand, the meta-analysis herein focuses on clinical reading results in a specific breast MRI indication subgroup and thus provides a more representative estimate of what is to be expected in clinical practice. This meta-analysis, therefore, provides a much need complement of the currently available meta-analytic literature on breast MRI focusing on other clinical indications such as preoperative staging [5], high-risk screening [6] and preoperative systemic therapy assessment [51,52].

As detailed in the introduction, there are various reasons to assigning conventional findings as equivocal. In its 5^th^ and most recent edition of the BI-RADS atlas, the ACR lexicon states that breast MRI is not an appropriate follow-up measure for minimal or equivocal findings [53]. Current European EUSOMA recommendations stipulate that MRI should not be used as a problem solving tool if percutaneous biopsy can be performed while the less specific EUSOBI guidelines list problem-solving as an MRI indication [50,54]. In addition to the results of the already discussed general meta-analysis on breast MRI, the reticence to routinely use breast MRI as a problem-solving tool stems at least in part from the concern of whether conclusions derived from data obtained in one institution are pertinent to other institutions that may have applied somewhat different problem-solving indications, used different imaging equipment, or catered to different populations, to cite a few variables that can impact findings. The substantial variations in cancer prevalence identified by our analysis, ranging between 2–56%, confirms this concern and calls for a better definition of problem solving criteria. This finding is backed up by our QUADAS 2 assessment, identifying high or unclear risk of bias regarding patient selection in a number of studies. In particular, we found problem-solving indications rather generally defined in the inclusion criteria of the investigated studies while the majority did not stratify the reported results regarding the specific findings that led to breast MRI. Meta-regression unvealed a tendency towards higher sensitivity in larger studies and studies with a higher prevalence of malignancy. This could be due to higher reader experience in more experienced centers with a higher patient throughput and possibly also a better patient preselection were patients with a higher chance of malignancy were referred to MRI. On the other hand, this effect could be attributed to the clear outlier study by El-Barhoun and Pitman [18], as removal of their data eliminated statistical significance.

Regarding specificity, meta-regression found significantly higher values for studies focusing on BI-RADS 0 and 3 lesions as compared to all other indications. However, this is based only on two out of 14 studies and although the effect is significant, the lack of data on lesion presentation in conventional imaging (e.g. architectural distortion vs solid lesion) clearly warrants further exploration. Therefore, due to the lack of indication-based subgroup data, this meta-analysis does not provide conclusions on adequate or inadequate indications for problem solving MRI. It appears reasonable to assume an only minor oncologic value of additional breast MRI in case of very low pre-test probabilities after negative conventional workup. In other words, our metaanalysis-analysis indicates that breast MRI ought not to be used to simply confirm negative conventional imaging.

This study has several limitations. First, it did not address the financial concerns that may be associated with the more frequent use of breast MRI, a subject that is best discussed within the framework of affordable healthcare for cancer patients [55]. It is however worth mentioning that recent indicators show that breast MRI costs are becoming reasonably low in some settings [56,57]. The recent increase in utilization of breast MRI has substantially expanded the data on breast MRI used as a problem-solving tool, which among other things enabled this study. Second, between-study heterogeneity was high, a finding in line with—if not in particular caused by—the highly variable prevalence of malignancy. As detailed elsewhere, PPV and NPV are affected by disease prevalence. While PPV positively correlates with prevalence, NPV negatively associates with it. Our results show a strong variation of PPV between studies (26%-96%), while NPV ranged only from 98% to 100% in thirteen out of fourteen studies. The remaining outlier had an NPV of 88%. Bivariate meta-analysis considers sensitivity and specificity as dependent variables. Consequently, a high heterogeneity on one of these parameters affects the other. This holds true for our moderately high PPV findings that do reflect the underlying heterogeneity in study populations as evidenced by their prevalence of malignancy. Conversely, the NPV of the studies included in our meta-analysis stayed robust over a wide range of prevalence values, a finding due to two reasons: high sensitivity and relatively low prevalence of malignancy. The high heterogeneity should thus not be seen as a limitation but rather a strength of our analysis: the robust and high NPV of breast MRI in the investigated setting underlines that MRI can exclude breast cancer with high accuracy despite the observed heterogeneity of problem-solving indications. Third, and as outlined above, due to a lack of indication-based subgroup data, this meta-analysis does not provide data on adequate or inadequate indications for problem solving MRI.

## Conclusion

According to our results, breast MRI demonstrates an excellent diagnostic performance in case of non-calcified equivocal breast findings detected at conventional imaging. However, considering the substantial heterogeneity regarding prevalence of malignancy, problem-solving criteria need to be better defined.

## Supporting Information

S1 Fig QUADAS 2 graph illustrating the results of risk of bias and applicability assessment. (PNG)

S2 Fig sROC curve. (TIFF)

S1 File Meta-Regression analysis on factors influencing sensitivity and specificity. (DOCX)

S1 Table PRISMA 2009 Checklist. (DOC)

S2 Table Technical details on MRI studies included in meta-analysis. (*) unless otherwise specified; NA: Not applicable.
(DOCX)
