# Supplementary figures and images for: Diagnostic Performance of Breast Magnetic Resonance Imaging in Non-Calcified Equivocal Breast Findings: Results from a Systematic Review and Meta-Analysis
Source: PLoS One. 2016 Aug 2;11(8):e0160346. doi: 10.1371/journal.pone.0160346 (PMC4970763; doi:10.1371/journal.pone.0160346)

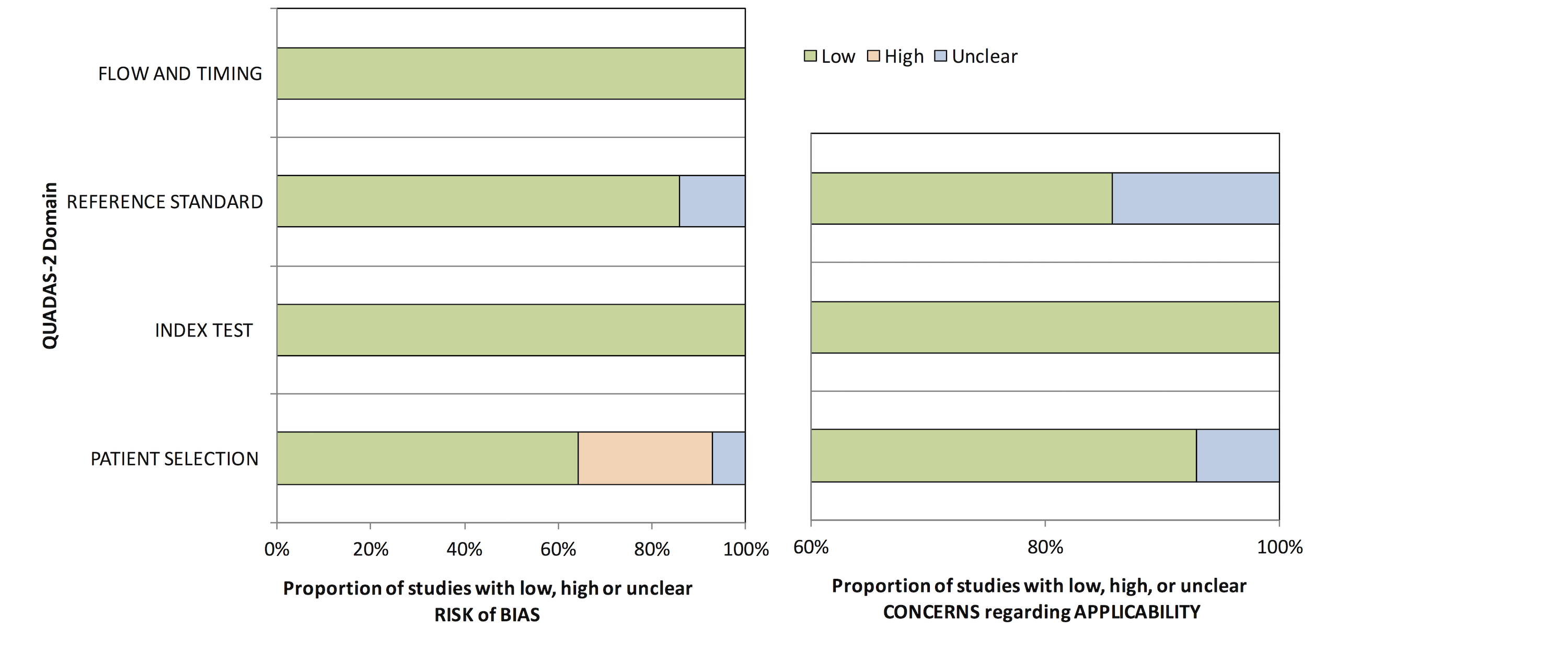

Supplement: S1 Fig — (PNG) [file pone.0160346.s001.png]

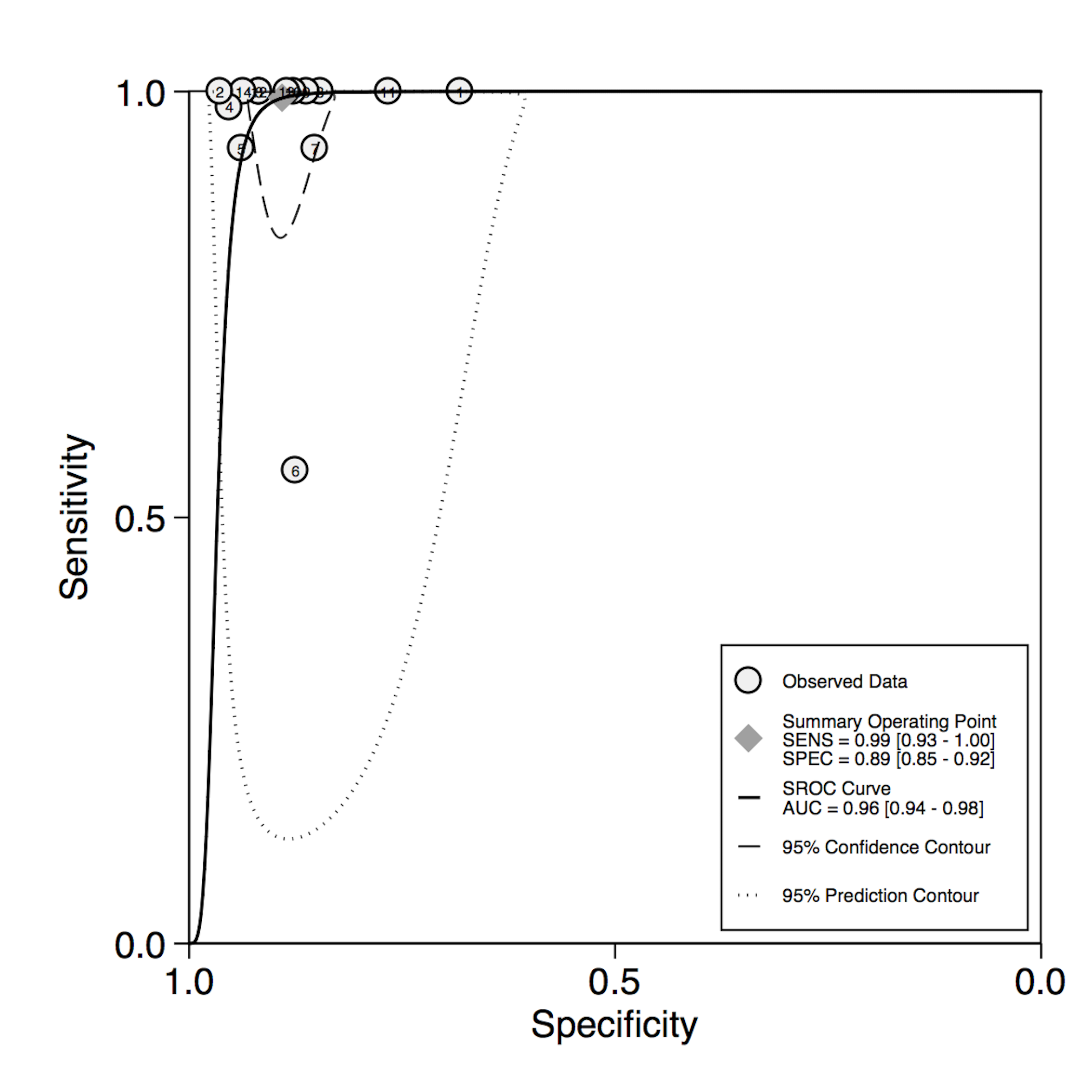

Supplement: S2 Fig — (TIFF) [file pone.0160346.s002.tiff]
