# Supplementary material for: Diagnostic Performance of Breast Magnetic Resonance Imaging in Non-Calcified Equivocal Breast Findings: Results from a Systematic Review and Meta-Analysis
Source: PLoS One. 2016 Aug 2;11(8):e0160346. doi: 10.1371/journal.pone.0160346 (PMC4970763; doi:10.1371/journal.pone.0160346)
Supplement: S1 File — (DOCX) [file pone.0160346.s003.docx]

**Supplemental data**

Meta-regression results

Sensitivity, all studies:


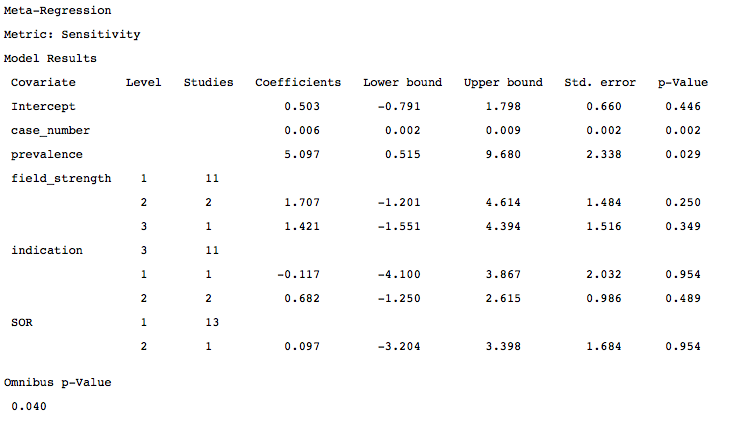


Sensitivity, excluding El-Barhoun 2011:


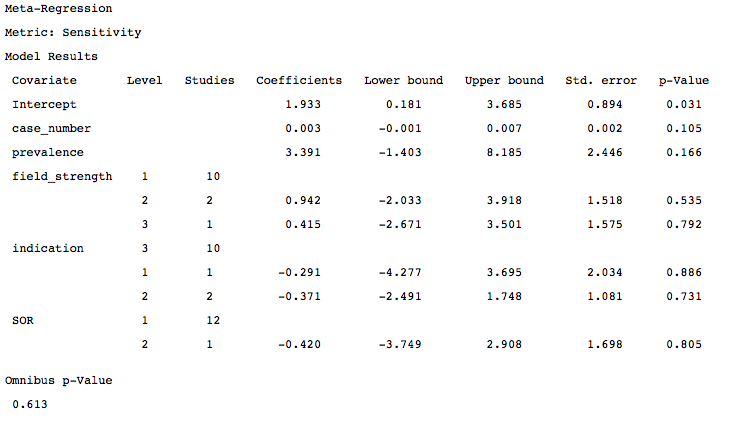


Specificity, all studies:


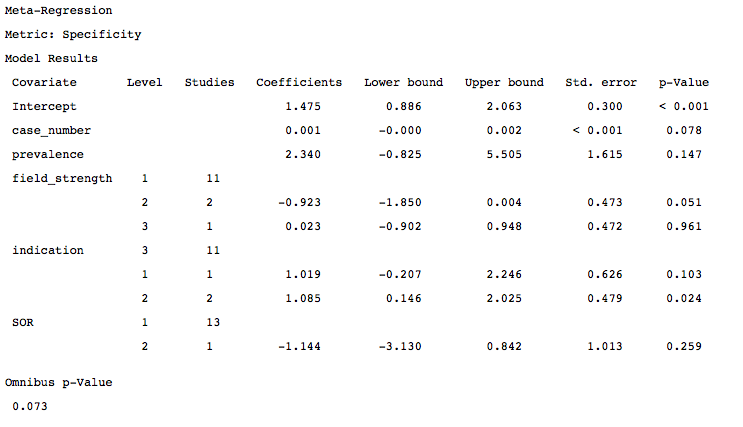


**Factor variable coding:**

Field strength: 1=1.5T, 2=1.5 or 3T, 3=3T

Indication: 1= BI-RADS 0 only, 2= BI-RADS 0 and/or 3, 3= not specified problem solving

SOR /standard of reference): 1= histopathology and follow-up, 2= histopathology only
