# Supplementary material for: Diagnostic Performance of Breast Magnetic Resonance Imaging in Non-Calcified Equivocal Breast Findings: Results from a Systematic Review and Meta-Analysis
Source: PLoS One. 2016 Aug 2;11(8):e0160346. doi: 10.1371/journal.pone.0160346 (PMC4970763; doi:10.1371/journal.pone.0160346)
Supplement: S2 Table — (*) unless otherwise specified; NA: Not applicable. (DOCX) [file pone.0160346.s005.docx]

| **First author, year** | **MRI field strength** | **Contrast agent dosage (mmol/kg)*** | **Contrast agent** | **Contrast agent**  **injection rate** | **Fat saturation in DCE** |
| --- | --- | --- | --- | --- | --- |
|  |  |  |  |  |  |
| **Lee,1999** | 1.5 | 0.1 | No details | NA | Both |
| **Gökalp, 2006** | 1.5 | 0.1 | Gadopentetic acid | NA | No |
| **Moy, 2009** | 1.5 | 0.1 | Gadopentetic acid | 2 ml/s | Yes |
| **Pediconi, 2009** | 1.5 | 0.1 | Gadobenate dimeglumine | 2 ml/s | No |
| **Benndorf, 2010** | 1.5 | 0.1 | Gadopentetic acid | 3 ml/s | No |
| **El-Barhoun, 2011** | 1.5 | 0.16 | Gadopentetic acid | NA | Yes |
| **Yau, 2011** | 1.5 | 0.1 or 20 ml | Gadodiamide | Manual or 2 ml/s | Yes |
| **Dorrius, 2012** | 1.5 | 0.1 | Gadoterate meglumine | NA | No |
| **Lobrano, 2012** | 3 | 20 ml | No details | NA | NA |
| **Olsen, 2012** | 1.5 | 0.1 | No details | 3 ml/s;  manual before 2005 | Yes |
| **Bick, 2013** | 1.5 or 3 | Single or double dose | No details | NA | No |
| **Oztekin, 2014** | 1.5 | 0.2 | No details | NA | No |
| **Spick, 2015** | 1.5 or 3 | 0.1 | Gadoteridol | NA | No |
| **Strobel, 2015** | 1.5 | NA | No details | NA | No |

**Supplemental Table 1.**
